# Detection and molecular characterization of porcine reproductive and respiratory syndrome virus in Lithuanian wild boar populations

**DOI:** 10.1186/s13028-016-0232-5

**Published:** 2016-09-08

**Authors:** Arunas Stankevicius, Jurate Buitkuviene, Virginija Sutkiene, Ugne Spancerniene, Ina Pampariene, Arnoldas Pautienius, Vaidas Oberauskas, Henrikas Zilinskas, Judita Zymantiene

**Affiliations:** 1 Faculty of Veterinary Medicine, Lithuanian University of Health Sciences, Tilzes st. 18, LT-47182 Kaunas, Lithuania; 2 National Food and Veterinary Risk Assessment Institute, J. Kairiukscio st. 10, LT-08409 Vilnius, Lithuania

**Keywords:** PRRSV, Wild boar, RT-PCR, Real-time RT-PCR, Virus prevalence, ORF5 sequences

## Abstract

**Background:**

Porcine reproductive and respiratory syndrome virus (PRRSV) is recognized worldwide as an important and economically devastating pathogen in pig production. Although PRRSV is widespread in domestic swine, there is a lack of information regarding PRRSV infection in European wild boars (*Sus scrofa*). Currently available information does not provide conclusive evidence that wild boars are a reservoir of PRRSV. Nevertheless, wild boars may be likely to become infected by domestic swine through occasional direct or indirect contact. Furthermore, wild boars can act as a reservoir for infectious diseases of domestic pigs. Therefore, the objectives of the present study were to determine the virus prevalence and further explore the epidemiology and diversity of PRRSV strains present in Lithuanian wild boars over a 5-year period. A total of 1597 tissue and serum samples from wild boars inhabiting 44 districts and ten counties in Lithuania were analysed using conventional nested reverse transcription polymerase chain reaction (RT-PCR) and real-time Taqman RT-PCR for the detection of PRRSV-specific open reading frame (ORF) 1 and 6 sequences.

**Results:**

PRRSV was highly prevalent in Lithuanian wild boar populations, with an average rate of 18.66 % using conventional RT-PCR and 19.54 % using real-time RT-PCR. PRRSV was detected in 36.71 and 41.77 % of 237 hunting grounds tested by conventional RT-nPCR and real-time RT-PCR, respectively. No statistically significant differences in PRRSV prevalence were observed by geographic area in the ten Lithuanian counties. Animals infected with PRRSV were identified in all age groups; however, significantly higher prevalence rates were identified in subadult and adult wild boars than in juveniles up to 12 months old. No positive results were obtained using conventional PCR with Type 2 specific primers. Phylogenetic analysis of the partial ORF5 region revealed that ten wild boars harboured virus sequences belonging to genetic subtypes 3 and 4 and may therefore pose a serious threat to Lithuanian pig farms in which only subtype two strains are circulating.

**Conclusions:**

The results of virus prevalence and phylogenetic analyses strongly support the role of wild boars as a possible natural reservoir for PRRSV in Lithuania.

**Electronic supplementary material:**

The online version of this article (doi:10.1186/s13028-016-0232-5) contains supplementary material, which is available to authorized users.

## Background

Porcine reproductive and respiratory syndrome virus (PRRSV) is globally regarded as an important and economically devastating pathogen in pig production characterized by respiratory disease in piglets and reproductive failure in sows. PRRSV, a member of the family *Arteriviridae* in the order *Nidovirales,* is a small, enveloped virus with a single-stranded positive-sense RNA genome approximately 15 kb in length that encodes at least nine open reading frames (ORF), including ORF1a, 1b, 2a, 2b, and 3–7 [1, 2]. ORFs 2a, 2b, 3, 4, and 5 encode envelope glycoproteins, while ORFs 6 and 7 encode the matrix and nucleocapsid proteins, respectively. The largest and most conserved genes are ORF1a and ORF1b, which encode the viral RNA polymerase. ORF5 encodes the major envelope protein and is often used for phylogenetic analysis and molecular characterization, mainly because of its high variability and large number of available sequences [3, 4]. A novel PRRSV ORF5a protein encoded in an ORF that overlaps the major envelope glycoprotein GP5 ORF has recently been identified [5], and a—two ribosomal frame-shifting has recently been identified for the expression of nonstructural proteins nsp2TF in the nsp2-coding region. The nsp2TF coding sequence is conserved in the PRRSV genome [6].

Based on genetic differences, PRRSV has been divided into two genotypes: Type 1, mainly comprising viruses from Europe, and Type 2, mainly comprising of viruses from North America and Asia. The two types are 55–70 % identical at the nucleotide level. These two PRRSV genotypes have emerged almost simultaneously on their respective continents since the late 1980s. Publications describing the ORF5 PRRSV sequences have shown that the genetic diversity of Type 1 is higher than that of Type 2 [7–9]. A unique cluster of Type 1 PRRSV was thought to be closely related to the common ancestors of the European and American strains was detected in Lithuania [10]. Investigations in ORF5 and ORF7 regions of PRRSV conducted in Belarus and Russia have shown that nucleotide sequences in virus isolates from these countries also differ significantly from those in PRRSV strains circulating in Western Europe [8, 9]. Based on ORF5 and ORF7 sequences, Type 1 East European PRRSV strains were divided into four genetic subtypes representing PRRSV strains prevalent in Belarus, Lithuania and Latvia [11].

Although PRRSV is widespread in domestic swine, there is a lack of information regarding PRRSV infection in European wild boars (*Sus scrofa*). The seroprevalence of antibodies against PRRSV in wild boars has been determined to range from 0.3 to 3.6 % in several countries [12–19]; however, in the Campania Region of Italy, a seroprevalence of 37.8 % was detected [17]. Many other studies have reported negative PRRSV seroprevalence results [20–24]. PRRSV has also been detected using reverse transcription polymerase chain reaction (RT-PCR) methods in the lung tissue of wild boars in Italy [25], Germany [26] and Lithuania [27] as well as in the lung tissue of hybrid wild boars, known as “special wild pigs” in China [28].

Currently available information does not provide conclusive evidence that wild boars are a reservoir of PRRSV [26, 29]. Nevertheless, wild boars may be likely to become infected by domestic swine through occasional direct or indirect contact. Furthermore, wild boars have been found to act as a reservoir for other infectious diseases of domestic pigs, and interactions between wide and domestic pig populations can potentially result in transmission of these diseases [13, 29]. In this case, PRRSV transmission would be favoured within dense wild boar populations, but the lack of infection in many wild boar populations in various European countries suggest that the initial transmission from domestic swine to wild boar does not occur or occurs very sporadically. Therefore, the objectives of the present study were to determine virus prevalence and further explore the epidemiology and diversity of PRRSV strains prevalent in Lithuanian wild boars over a 5-year period.

## Methods

### Wild boar samples

Samples were collected from wild boars (n = 1597) hunted in forested areas (21,740 km^2^) of all 44 districts and 10 counties of Lithuania during the 2011–2015 hunting seasons. Wild boars were numbered and categorized according to age (teeth method) and weight into three age groups: juveniles (n = 335), subadults (n = 652) and adults (n = 610). Lung (n = 755), lymph node and tonsil (n = 264), spleen (n = 143), or serum (n = 435) samples were collected from hunted wild boars within 2–3 h after death from public or private hunting grounds (n = 237) and stored at −20 °C until analysis.

### Pig samples

Lung samples from dead weaned pigs (n = 32) were collected from PRRSV-positive farms (n = 5) located near the sites where wild boars were shot. RNA was obtained from PRRSV-positive samples, and ORF5 sequences were used for phylogenetic analysis. All lung samples were transported at 5 °C and then stored at −20 °C until analysis.

### RNA isolation and cDNA synthesis

RNA was extracted from tissue samples using the GeneJET RNA purification kit (Thermo Fisher Scientific, Waltham, USA). For each extraction, 30–50 mg of tissue sample was ground thoroughly with a mortar and pestle. Lysis buffer (300 µl) supplemented with β-mercaptoethanol was added. The remaining steps were performed following the manufacturer’s instructions. Extracted RNA was eluted in 100 µl nuclease-free water. Total RNA was extracted using the GeneJET Viral DNA and RNA Purification Kit (Thermo Fisher Scientific), designed for rapid and efficient purification of high quality viral nucleic acids from various human and animal liquid samples such as plasma, serum, whole blood. Wild boar serum (200 µl) was used for RNA extraction according to manufacturer protocol. Extracted RNA samples were stored at −80 °C until further analysis.

Reverse transcription (RT) was performed on extracted RNA. Five microlitre RNA was mixed with 1 µl Oligo(dT)18 primer (Thermo Fisher Scientific); 6.5 µl DEPC-treated water; 4 µl 5× reaction buffer (Thermo Fisher Scientific); 0.5 µl (20 U) Thermo Scientific RiboLock RNase Inhibitor; 2 µl dNTP Mix (10 mM each); and 1 µl (200 U) RevertAid reverse transcriptase (Thermo Fisher Scientific). A total volume of 20 µl reaction mixture was incubated for 60 min at 42 °C, and the reaction was then terminated by heating at 70 °C for 10 min. The obtained cDNA was then used for PCR and real-time PCR.

### PCR and real-time PCR

A 25 µl PCR mixture containing 5 µl cDNA; 1× Taq polymerase reaction buffer (Thermo Fisher Scientific); 2.5 mM MgCl_2_; 0.2 mM dNTP Mix; 0.6 U Taq polymerase (Thermo Fisher Scientific); and 20 pmol of each primer was used for amplifying ORF1 258 bp sequences [26] (see Additional file 1). According to a previous study [26], conventional RT-PCR targeting ORF1 has been performed to detect the Type 1 or Type 2 PRRSV in wild boar samples. PCR primers were designed based on ORF 1b and found to be more conserved within and between the two PRRSV virus genotypes than those of other genes.

The nested PCR contained the same reagents as the first PCR except primers were used to amplify ORF1 186 bp sequences for Type 1 PRRSV and 108 bp sequences for Type 2 PRRSV strains [26] (see Additional file 1) and 2.5 µl of the PCR product was used as a template for the nested PCR assay. The positive samples in the ORF1 RT-nPCR and ORF6 real-time RT-PCR were further analysed by amplifying the ORF5 sequences used for phylogenetic analysis of PRRSV. For the ORF5 region, amplification PCR and nested PCR in a final volume of 25 µl were performed using 20 pmol of each primer specific for this region [10]. Details of primers used are displayed in Additional file 1. All reactions were performed in a Mastercycler personal thermocycler (Eppendorf, Hamburg, Germany). Thermal cycling consisted of initial denaturation at 95 °C for 3 min, 40 amplification cycles of 95 °C for 30 s, annealing at 55 °C for 30 s and extension at 72 °C for 60 s followed by final extension at 72 °C for 10 min. For ORF5 region amplification, thermal cycling was performed using 35 cycles of 94 °C for 60 s, 55 °C for 60 s and 72 °C for 90 s with final extension at 72 °C for 10 min. The nested PCR product was separated in 1.5 % agarose gel and visualized with UV light after ethidium bromide staining.

As an alternative to conventional RT-nPCR, real-time RT-PCR was performed using ORF6 region primers and a probe coding for the conserved structural membrane protein M [30]. The 25 µl real-time RT-PCR mixture consisted of 8.5 µl nuclease-free water; 12.5 µl TaqMan Universal Master Mix II with UNG (Applied Biosystems, Foster, USA); 1.0 µl each of the forward and reverse primers (20 µM), 1 µl probe (10 µM) (see Additional file 1); and 2.5 µl cDNA template. Real-time RT-PCR was performed with StepOnePlus (Applied Biosystems) Thermal Cycler using the following program: UNG incubation at 50 °C for 2 min; initial incubation at 95 °C for 10 min; and 40 cycles of 95 °C for 15 s and 60 °C for 60 s.

### Sequencing and phylogenetic analysis

Positive ORF5 nested PCR products were excised from the gels, purified using a GeneJET PCR Purification Kit (Thermo Fisher Scientific) and sequenced in both directions using the BigDye Terminator Cycle Sequencing Kit v3.1 (Applied Biosystems) and 3130× Genetic analyzer (Applied Biosystems). Sequences from both strands of the ORF5 PCR products were determined using the same primers used for nested PCR amplification. The sequences were submitted to Genbank under accession numbers KT828652-KT828665.

The obtained partial ORF5 sequences were compared with the reference set of sequences selected from GenBank to represent a full range of genetic diversity and geographic locations of Type 1 PRRSV. The sequences were aligned using the Clustal W software from MegAlign (Lasergene software package, DNASTAR Inc, Madison, USA). Bootstrap values were calculated using CLC Gene Free Workbench software, with bootstrap values based on 100 analysis replicates (v4.0.01, CLC bio A/S, Aarhus, Denmark).

### Statistical analysis

Descriptive statistics were calculated using Microsoft Excel 2007 and IBM SPSS Statistics (Version 21.0). Z-tests for proportions were used to estimate the apparent prevalence confidence intervals (95 % CI), and χ^2^-tests for equality of two proportions were used to determine significant differences in prevalence between sampling periods, age groups, and counties. The results were considered statistically significant if P values were <0.05.

## Results

A total of 1597 samples (lung, lymph node, tonsil, spleen or serum samples) from wild boars inhabiting 44 districts and 10 counties in Lithuania were analysed using conventional nested RT-PCR and real-time Taqman RT-PCR for detection of PRRSV-specific ORF1 and ORF6 sequences, respectively. PRRSV was detected in 18.66 % (298/1597) of wild boars tested using RT-nPCR and 19.54 % (312/1597) of samples tested using real-time RT-PCR (Table 1). Differences in PRRSV prevalence during the sampling period (2011–2015) were not significant (P > 0.05) irrespective of PCR method.

**Table 1:** Data from RT-nPCR and real-time RT-PCR assays of wild boar samples obtained from 2011 to 2015

Sampling year	Number of hunting grounds tested	Number of wild boars tested	RT-nPCR			Real-time RT-PCR		
			Number of positive wild boars	Percentage of positive wild boars	95 % confidence interval (%)	Number of positive wild boars	Percentage of positive wild boars	95 % confidence interval (%)
2015	37	187	32	17.11	11.71–22.51	34	18.18	12.65–23.71
2014	55	268	43	16.04	11.65–20.43	47	17.54	12.99–22.09
2013	45	290	52	17.93	13.52–22.34	54	18.62	14.14–23.10
2012	52	489	101	20.65	17.06–24.24	104	21.27	17.64–24.90
2011	48	363	70	19.28	15.22–23.34	73	20.11	15.99–24.23
Total	237	1597	298	18.66	16.75–20.57	312	19.54	17.60–21.48

**Fig. 1:**
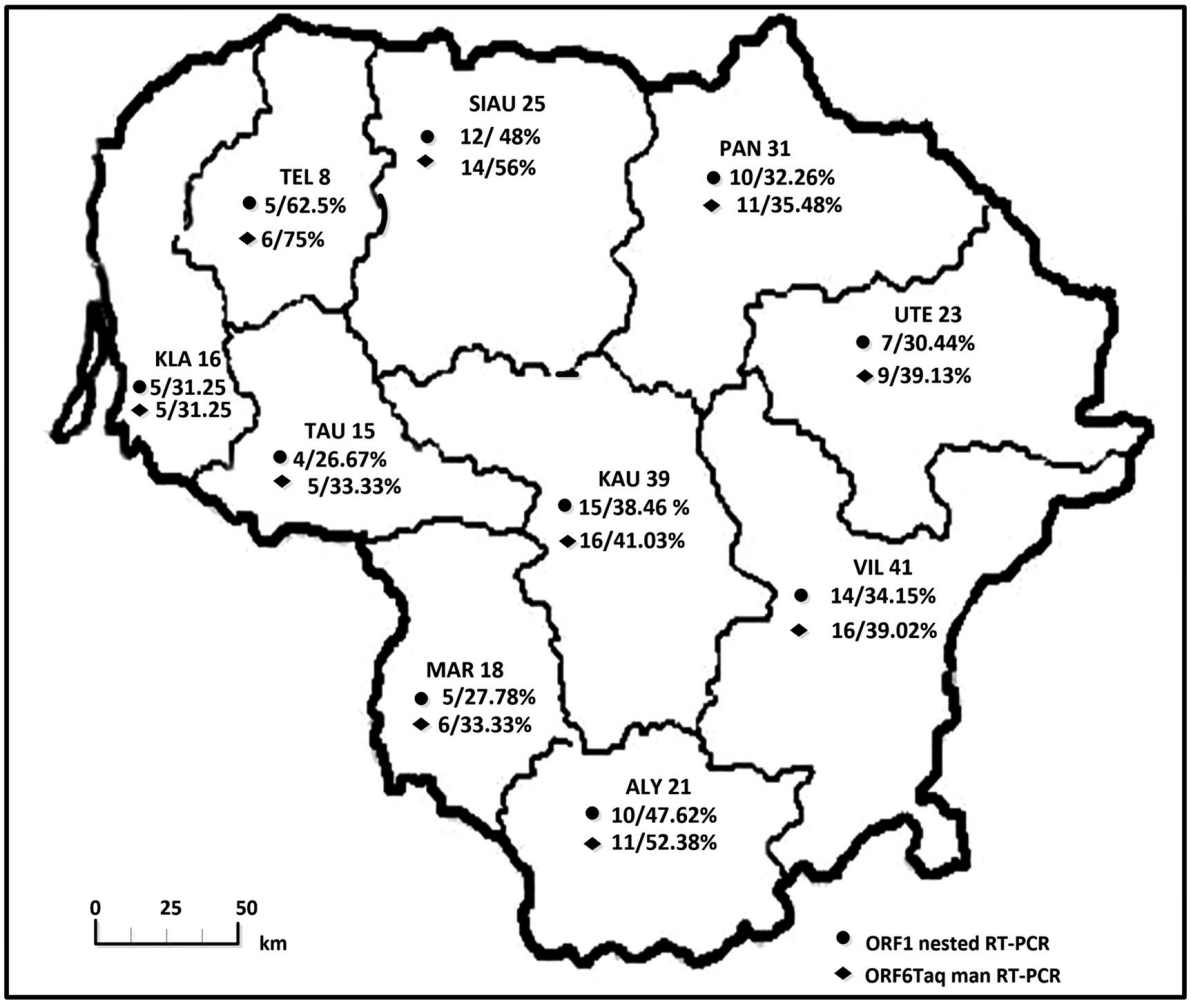
PRRSV prevalence distribution by hunting grounds in different Lithuanian counties. *Bold letters* indicate counties: *ALY* Alytus, *MAR* Marijampole, *VIL* Vilnius, *KAU* Kaunas, *TAU* Taurage, *KLA* Kaipeda, *TEL* Telsiai, *SIAU* Siauliai, *PAN* Panevezys, *UTE* Utena. The* numbers* indicate tested hunting grounds in each county. *Percentage in the second line* indicates prevalence rate determined by nested and real-time RT-PCR

PRRSV Type 1-specific amplicons were detected with both RT-PCR methods in all 10 Lithuanian counties and 36 of 44 districts (data not shown). PRRSV was detected in 87 (36.71 %; 95 % CI 30.57–42.85 %) and 99 (41.77 %; 95 % CI 35.49–48.05) of the 237 hunting grounds tested by conventional RT-nPCR and real-time RT-PCR, respectively (Fig. 1). The highest PRRSV prevalence was detected in Telsiai County at 62.5 % (95 % CI 28.95–96.05 %) by RT-nPCR and 75 % (95 % CI 44.99–105.01 %) by real-time RT-PCR. The differences between PRRSV prevalence by geographic area in all ten Lithuanian counties were also not significant (P > 0.05) irrespective of PCR method.

The PRRSV prevalence for different age groups of wild boars is presented in Table 2. Animals infected with PRRSV were found in all age groups; however, the highest prevalence rates were found in adults and subadults (Table 2). Subadults and adults were twice as likely to be PCR positive than the juvenile boars (P < 0.05).

**Table 2:** Prevalence of PRRSV infection in wild boars detected by nested and real-time RT-PCR by age group

Age group	Number of wild boars tested	RT-nPCR			Real-time RT-PCR		
		Number of positive wild boars	Percentage of positive wild boars	95 % confidence interval (%)	Number of positive wild boars	Percentage of positive wild boars	95 % confidence interval (%)
Juveniles (up to 12 months)	335	38	11.34	7.94–14.74	39	11.64	8.21–15.07
Subadults (12-24 months)	652	139	21.32	18.18–24.46	145	22.24	19.05–25.43
Adults (over 24 months)	610	121	19.84	16.68–23.00	128	20.98	17.75–24.21
Total	1597	298	18.66	16.75–20.57	312	19.54	17.60–21.48

PRRSV Type 2 was not detected using conventional RT-nPCR with ORF1-specific primers in 1597 tested wild boars from 237 hunting grounds.

**Fig. 2:**
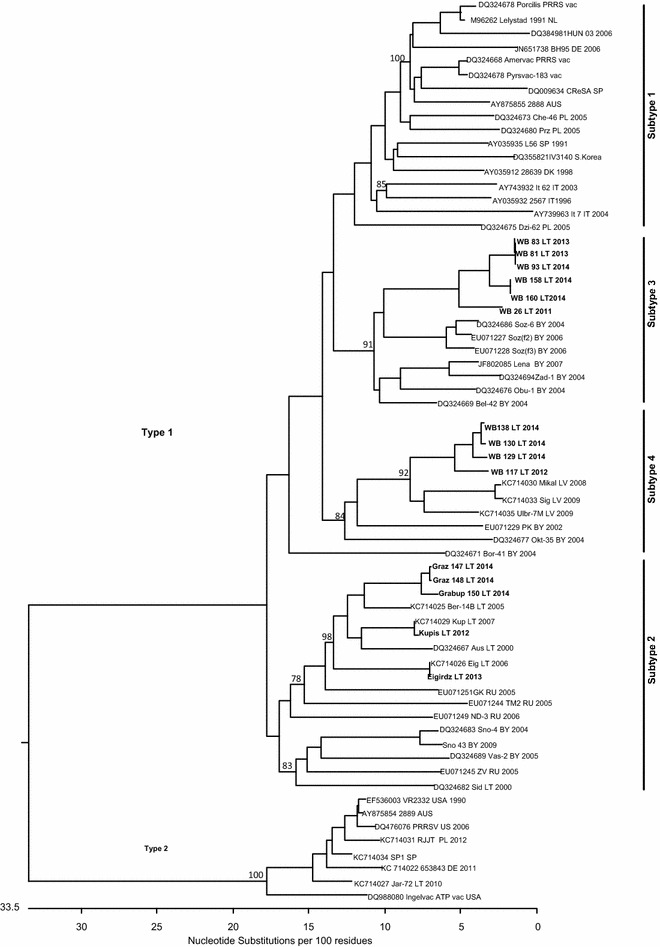
Phylogenetic analysis of Lithuanian wild boar ORF5 sequences. Clustal W algorithm was used for sequence alignment.* Numbers* adjacent to main branches indicate bootstrap values for different genetic subtypes within the European type of PRRSV. The reference sequences are marked as follows: Gen Banks Access No., name of the sequence, country (up to three letters sign), and years of detection. Sequences determined in this study are indicated in *bold* (GeneBank accession numbers KT828652-KT828665)

For genetic comparison of circulating PRRSV strains in Lithuanian wild boars, ten amplification products of partial ORF5 region were sequenced. All obtained sequences showed the highest similarity to PRRSV Type 1 sequences. Phylogenetic analysis of the partial ORF5 region revealed that wild boar sequences belonged to genetic subtypes 3 and 4 (Fig. 2). The wild boar PRRSV sequences formed well-defined clusters within these subtypes and were aligned with PRRSV ORF5 published reference sequences from domestic pigs in Belarus and Latvia. Interestingly, these subtypes have never been detected in domestic pigs in Lithuania. ORF5 sequences obtained from Lithuanian pig farms clustered in subtype 2 of the phylogenetic tree along with reference sequences previously obtained from Lithuanian, Belarus and Russian Federation pig farms.

## Discussion

The study shows that PRRSV infections are prevalent in Lithuanian wild boar populations with an average detection rate of 18.66 % using conventional ORF1 RT-nPCR and 19.54 % tested using real-time RT-PCR. This proportion appears to be quite higher than that indicated in a previous investigation, which found that PRRSV by RT-nPCR was detected in 15.9 % of wild boar samples in Germany [26]. Surveys of wild boars from eastern Slovakia have revealed that PRRSV was present in 1.6 % of samples when tested by nested RT-PCR [31], and PRRSV Type 1 was accidentally identified in a road killed wild boar in Italy [25]. Contrary to our results, Kukushkin et al. [20] failed to detect PRRSV in tissue samples from wild boars in Russia using RT-PCR, while a study in Poland found that PRRSV infections were not prevalent in wild boars [32]. The sera and tissues from wild boars in south-central Spain were also found to be negative by conventional and real-time RT-PCR assays [18].

Throughout Lithuania, the prevalence of PRRSV infection was higher in wild boars from hunting grounds (36.71 and 41.77 % depending on PCR used) than in the general porcine population. The presence of PRRSV-positive wild boars in all Lithuanian counties may be explained by the favourable conditions for wild boars that have developed throughout Lithuania. The population density of wild boars in Lithuanian forests has increased considerably from 1.84 wild boars per km^2^ in 2011 to 2.66 wild boars per km^2^ in 2015 [33]. Furthermore, these findings could be explained by migration of wild boars from neighbouring countries and their ability to colonize new habitats through abundant supplementary feeding. Supplementary feeding of wild boars during winter has been practised in Lithuania for many years as a dissuasive measure aimed to reduce crop damage by wild boars or an attractive measure during hunting season. Supplementary feeding brings animals closer together near feeding locations, leading to increased level of aggregation among and contact between wild boars. The results of our investigation revealed as unexpectedly high prevalence of PRRSV in wild boars; however, additional studies of wild boar populations in neighbouring Latvia, Belarus, and Kaliningrad Region of Russian Federation are necessary to investigate this further.

The highest prevalence of infected wild boars (19.84 to 22.24 %) was identified in the subadult and adult age groups, a finding that may be explained by an age-dependent higher risk of virus exposure.

This study demonstrated that wild boars can harbour different genetic lineages of PRRSV strains than those found in domestic pigs in Lithuania. This may pose a serious threat to the Lithuanian pig industry, where only subtype 2 strains are circulating. Contemporary investigations have found that subtype 3 strains identified in Belarus pig farms [8] may be highly virulent [34]. The most striking finding is detection of the subtype four strain in wild boars. Previously, this subtype had only been identified in pigs in Belarus and Latvia [8, 11].

In the present study, PRRSV ORF5 partial sequences were obtained only after amplification of highly ORF5 PCR-positive samples. Many ORF5 weak positive samples were not suitable for sequencing or resulted in sequences of poor quality. A possible explanation for this result might be a level of RNA copies in the samples that could only be detected by ORF1 RT-nPCR or by ORF6 real-time RT-PCR. Moreover, Reiner et al. [26] failed to amplify ORF5 as well as ORF7 sequences from wild boars with three PCR-systems that were applied in routine diagnostics of domestic pig samples.

The presence of different PRRSV subtypes in wild boars and pigs suggests that PRRSV infection may be an endogenous infection of wild boars that can serve as a reservoir for infection of domestic pigs. Wild boars have been identified as reservoirs for other viruses, such as those causing classical swine fever and Aujeszky’s disease [13]. Therefore, wild boars should be considered important source of viral infections in domestic pigs.

Detection of the highly diverse PRRSV subtypes 3 and 4 in Lithuanian wild boars may also indicate the emergence of PRRSV in domestic pigs. Shi et al. [4] suggested that ancestors of PRRSV subtype 3 may have been present in Eastern Europe before the emergence of subtype 1 PRRSV in Western European pig farms. By molecular clock analysis, the most recent common ancestor for PRRSV Types 1 and 2 existed at least 100 years ago [35]. Although it is possible that PRRSV diverged from other arteriviruses, the pre-emergence evolutionary history of this virus remains a mystery. If wild boars had a longer history of hosting PRRSV strains than domestic pigs, greater viral diversity in wild boars would also be expected. PRRSV ORF5 partial sequences from wild boars obtained in this study exhibited levels of diversity similar to findings in domestic swine population in Lithuania, Latvia, Belarus and European and Asian regions of the Russian Federation [8–10] but different from subtype 1 strains circulating in Central and Western Europe and worldwide. The exceptionally high diversity of PRRSV ORF5 in Eastern Europe indicates that this genotype was established there before establishment in Western Europe; a finding that favours the hypothesis that PRRSV Type 1 emerged in Eastern Europe [4, 6]. Phylogenetic analyses of ORF1 viral sequences from wild boars in Germany [26] presented two highly homologous groups clustered within the diversity of PRRSV Types 1 and 2; however, amplification of ORF5 or ORF7 sequences was not successful. ORF5 encodes the major envelope protein and is often used for phylogenetic analyses mainly because of its high variability; therefore, it has been proposed for subtype definition of PRRSV Type 1 strains [11].

## Conclusions

Wild boars may act as a natural reservoir for PRRSV in Lithuania. PRRSV was highly prevalent in Lithuanian wild boar populations, with an average prevalence rate of 18.66 % using conventional RT-PCR and 19.54 % using real-time RT-PCR. PRRSV was detected in 36.71 and 41.77 % of 237 hunting grounds tested by conventional RT-nPCR and real-time RT-PCR, respectively. Phylogenetic analysis of the partial ORF5 region revealed that 10 wild boars harboured virus sequences belonging to genetic subtypes 3 and 4 and may therefore pose a serious threat to Lithuanian pig farms in which only subtype 2 strains are circulating.

## Additional file

**Additional file 1.** Primers and probe used for the conventional and real-time PCRs to detect PRRSV [10, 26, 30].
