# Supplementary material for: Detection and molecular characterization of porcine reproductive and respiratory syndrome virus in Lithuanian wild boar populations
Source: Acta Vet Scand. 2016 Sep 8;58:51. doi: 10.1186/s13028-016-0232-5 (PMC5016999; doi:10.1186/s13028-016-0232-5)
Supplement: Supplementary file 1 — Additional file 1. Primers and probe used for the conventional and real-time PCRs to detect PRRSV [10, 26, 30]. [file 13028_2016_232_MOESM1_ESM.docx]

# Additional Table Primers and probe used for the conventional and real-time PCRs to detect PRRSV

| Primer name | Primer type | Primer sequence (5’-3’) | PCR product size (bp) | Region amplified | PRRSV type |
| --- | --- | --- | --- | --- | --- |
| PRRSV1^a^ | Forward | CAACCTCCTGTATGAACTTGC | 258 | ORF1b | Common  type 1-2 |
| PRRSV2^a^ | Reverse | AGGTCCTCGAACTTGAGCTG |  |  |  |
| PRRSV3n^a^ | Forward | CTGTATGAACTTGCAGGATG | 186 | ORF1b | Type1 |
| PRRSV4n^a^ | Reverse | CGACAATACCATGTGCTG |  |  |  |
| PRRSV NA1^a^ | Forward | GGCGCAGTGACTAAGAGA | 108 | ORF1b | Type 2 |
| PRRSV NA2^a^ | Reverse | GTAACTGAACACCATATGCTG |  |  |  |
| EUORF5B^b^ | Forward | CAATGAGGTGGGCIACAACC | 606 | ORF5 | Type 1 |
| EUORF5C^b^ | Reverse | TATGTIATGCTAAAGGCTAGCAC |  |  |  |
| ORF5Fn^b^ | Forward | ATGAGATGTTCTCACAAATTGGGGCG | 552 | ORF5 | Type 1 |
| ORF5Rn^b^ | Reverse | CTAGGCCTCCCATTGCTCAGCCGAAGT |  | ORF5 |  |
| EU6-343^c^ | Forward | GTAGAAAGTGCTGCAGGTCTCCA | 119 | ORF6 | Type 1 |
| EU6-462^c^ | Reverse | CACGAGGCTCCGAAGTCCT |  |  |  |
| EU6-MGB^c^ | Probe | 6FAM-CTGTGAGAAAGCCCGGAC-NFQ-MGB |  |  |  |

^a^ From Reiner et al.[26]

^b^ From Stadejek et al.[10]

^c^ From Revilla-Fernandez et al.[30]
